# Universal or Targeted Antenatal Care for Immigrant Women? Mapping and Qualitative Analysis of Practices in Denmark

**DOI:** 10.3390/ijerph16183396

**Published:** 2019-09-13

**Authors:** Sarah Fredsted Villadsen, Hodan Jama Ims, Anne-Marie Nybo Andersen

**Affiliations:** 1 Section of Social Medicine, Department of Public Health, University of Copenhagen, 1165 Copenhagen, Denmark; hodanims@gmail.com; 2 Section of Epidemiology, Department of Public Health, University of Copenhagen, 1165 Copenhagen, Denmark; amny@sund.ku.dk

**Keywords:** health policy, antenatal care, maternal and child health, quality of care, migration, institutional practice

## Abstract

Inequity in immigrants’ health during pregnancy and childbirth has been shown. We studied the Danish regional organization of public midwifery-based antenatal care (ANC) for immigrant women to assess the strengths and weaknesses of organizing ANC as either universal or immigrant-targeted. A telephone survey in 2012 to all the Danish maternity wards (*n* = 20) was conducted. Semi-structured interviews with midwives providing targeted care (*n* = 6) were undertaken and characteristics of care were qualitatively analyzed, having the immigrant density of the facilities, the Danish ANC policy, and theories of cultural competence as the frame of reference. Six maternity wards were providing immigrant-targeted ANC. Targeted care implied longer consultations and increased attention to the individual needs of immigrant women. At these facilities, navigation in the health care system, body awareness, and use of interpreter services were key topics. The selection of women for targeted care was based on criteria (including names) that risk stigmatizing immigrant women. The arguments for not providing targeted care included that immigrant-targeted care was considered stigmatizing. Current universal care may overlook the needs of immigrant women and contribute to inequities. A strategy could be to improve dynamic cultural competencies of midwives, interpreter services, and flexibility of the care provision of the universal ANC system.

## 1. Introduction

Social and ethnic disparities are documented in pregnancy and childbirth outcomes in Europe, North America, and Australia [1,2]. Social, cultural, and language differences between health care providers and immigrants have been shown to affect the interaction, trust, and communication [3], which leads to reduced quality of care [4,5,6]. In Norway and Sweden, communication issues have been linked directly to suboptimal health care for immigrant women and increased perinatal death rates [7,8]. Increased cultural competence and immigrant-targeted care are recommended as strategies for improved health care for ethnic minority women, however evidence regarding how to implement this best is still lacking [9].

The term cultural competence has been debated, as health care providers have interpreted and used the term culture statically and simplistically [10]. It has been assumed that training of health care providers in the cultures from specific countries could improve the understanding between providers and users [11]. However, this has led to cultural archetypes of patients (for example “Mexican patients”), which is a misrepresentation as all cultures are dynamic and people from the same country of origin, of course, are very heterogeneous. Instead, techniques for a more person-centered dialogue are put forward to better grasp the diversity [12]. An important issue is that health professionals need to understand how they themselves represent a culture that is based in biomedicine and that for all humans, culture shapes thinking and behaviors, so self-reflection about how this affects their communication with patients is an important starting point [12]. Further, in response to the increased diversity, health systems need to consider how the individual needs of users are the result of interactions between cultural aspects, social position, and gender issues [13]. Not only are the competencies of the individual health care providers of importance for the interpersonal interactions and quality of care, but also the organization of care at system level [13]. However, how these aspects are handled in current clinical practice for pregnant women and whether immigrant-targeted care gives a better platform for ensuring person-centered care is not clear and needs debate and attention.

### 1.1. Antenatal Care in Denmark

Around 60,000 children are born annually in Denmark and in 2012, 15% of all live-born children had immigrant mothers, a number that increased to 19% in 2018 [14]. Increased risk of stillbirth, as well as infant and childhood mortality, has been documented for children of some of the largest immigrant groups when compared to children of Danish-born women [15,16] and likewise increased risk of growth restriction and preterm birth [17] and both lower and higher rates of acute cesarean section rates [18] have been found.

The Danish ANC is regulated according to the health law [19], where it is stated that all women with legal residency in Denmark have the right to free care concerning pregnancy and birth. The Danish Health Authority publishes national recommendations for ANC [20] and has the responsibility to inspect the care provision, while regional health authorities have the responsibility to manage and perform within this scope [21]. Consequently, the organization and practice differ from region to region. Women start ANC at their GP around gestational weeks 6–10. At this first encounter, the woman will be referred to her preferred delivery unit at a hospital (the far majority of births are in hospitals), from where, midwife-based ANC is coordinated. During the first visit, the general practitioner (GP) assesses the medical, social, and mental needs of the woman, to know whether she can follow the standard ANC or needs more extensive care (obstetric, other medical, or psycho-social), which in the national recommendation is labeled “differentiation of care”. The need for interpreter services is also assessed at this encounter. The standard level of care is three visits at the GP and six visits at the midwife up until 40 weeks of gestation (Table 1).

In the national recommendations for ANC, immigrant women are described as a vulnerable group, with risks related to vitamin D deficiencies, anemia, hemoglobinopathy, gestational diabetes, consanguinity, infectious diseases, and fear of obstetric intervention [20]. Further, communication problems, increased vulnerability of refugees and asylum seekers, and female genital cutting are mentioned as special issues. It is highlighted that the care provision should be based on an individual assessment of the needs of every woman, however general remarks are made, which state that the professionals should use interpreter services and allow for extra time, use a culturally sensitive approach in the communication (without explaining what that is), and explain the purpose of the ANC consultations [20]. 

The Danish ANC has been characterized as universal (all women are entitled to the same care), however, with increasing attention to identify and help women considered to be in special need [22]. Thus, introducing selective and targeted approaches and partly abolishing the universal welfare provision. From a previous mixed method needs assessment [9] we knew that some maternity wards, in the hope of addressing ethnic disparity, had organized immigrant-targeted programs, while other maternity wards had universal models of care. With targeted approaches, definitions of deviations from normality are required for identifying those in need, and the selection tool for how to identify immigrant women in special needs is controversial, as it cannot be justified to consider all pregnant, immigrant women as having extra needs.

### 1.2. Objectives

The general objective of this study was to discuss the strengths and weaknesses of organizing ANC for immigrant women in targeted and universal models by analyzing the public, midwife-based ANC for immigrant women in Denmark. Specific objectives were to map where immigrant-targeted and universal care programs existed, characterize targeted care, and assess how the organization of care correlated with the immigrant density at the maternity wards. The discussion of strengths and weaknesses of targeted or universal models had the Danish ANC policy and theories cultural competence as a frame of reference.

## 2. Material and Methods

### 2.1. Telephone Survey

All public maternity wards in Denmark were identified from the homepage of the Danish Midwife Association [23]. The administrative midwives were identified and included in a telephone survey performed from October to December 2012. A brief standardized questionnaire with open-ended questions was designed to assess whether a ward provided any special immigrant-targeted ANC, delivery, or postpartum care. We defined immigrant-targeted care as care where the women, at enrolment to midwife-based ANC, were selected to be seen by a specific midwife in a program that was specifically reserved for ethnic minority women, whatever definition of ethnicity that was used locally. Facilities with the enrolment of ethnic minority women into existing routine ANC were defined as universal.

If a facility provided immigrant-targeted care, the midwife conducting this service was contacted by telephone in 2013 and invited to a semi-structured interview with a focus on the midwives’ perceptions of the needs of the pregnant immigrant women, selection criteria for the targeted care, contents of the targeted care, potential challenges met in interactions with immigrant women, and the specific qualifications of the midwives. One of the respondents answered the questions by email. Answers from each maternity ward were typed and systematically summarized and open-ended questions were thematically categorized. The themes were discussed by the authors, who agreed on a line of data presentation and argumentation.

### 2.2. Immigrant Density at the Maternity Wards

The density of immigrants per maternity ward was obtained using data from the Birth Registry and Population Registry in Statistics Denmark. We obtained information on maternal origin and place of delivery for all deliveries in Denmark in 2010. A person had Danish origin if she had at least one parent who was both a Danish citizen and born in Denmark. Immigrants and descendants did not have one parent who was both born in Denmark and a Danish citizen. Immigrants were born abroad and descendants in Denmark. The study population was divided into mothers having Danish, Western, or non-Western countries of origin according to the classification by Statistics Denmark [24]. In total, 407 non-Western women had missing values on immigrant status (descendant or immigrant) and these were categorized as immigrants. Immigrant density was calculated as the proportion of immigrants among the mothers, who gave birth at the specific maternity wards in 2010.

### 2.3. Discussion of Targeted or Universal Models

In the discussion section of this article, we will contextualize the rationalities for providing targeted or universal care for immigrant women. The results will be analyzed in relation to the Danish national recommendations for ANC [24], which, in our analysis, is considered to be the national ANC policy, and further, the strengths and weaknesses of targeted care are discussed having the theoretical perspectives of cultural competence as reference.

### 2.4. Ethics

The participating midwives gave their informed consent and the study was conducted following the Declaration of Helsinki. According to the Danish legislation no ethnical clearance can be given by the Danish Scientific Ethics Committee to surveys and qualitative research, however, the study was reported to the National Data Protection Agency Id. No: 514-0289/19-3000.

## 3. Results

By the end of 2012, there were 24 public maternity wards in Denmark. The midwife-based ANC was organized and managed from central administrations at the maternity ward level (department of obstetrics). Some administrative units were in charge of more than one maternity ward, giving a total of 20 administrative units. All 20 administrative midwives agreed to participate. The ANC took place at ANC clinics at different geographical locations at the community level. 

In Table 2, the immigrant density estimated using all births in Denmark in 2010 by women of Danish origin and immigrant women is shown for operating birth facilities in 2012. Of these deliveries, 4% were by Western immigrant women, while 10% were by non-Western immigrant women. Births by descendant women are not shown by birthplace due to small numbers, however, Western descendant women had 140 deliveries in 2010, constituting 0.2% of the deliveries. The proportion was fairly even across facilities. Non-Western descendant women had 940 deliveries, constituting 1.6% of births, with a range of 0%–6% across the facilities.

### 3.1. Characteristics of Immigrant-Targeted ANC

Six of the 24 maternity wards had ANC clinics where immigrant-targeted ANC was provided (Table 2). None of these six facilities had written materials or evaluations of the targeted care, however, all of them reported more than five years of experience with targeted care. The densities of Western immigrants at these facilities (2%–7%) were around the total average of 4%, while the densities of non-Western women were lower (6%–7%) than the national average of 10% at all six, but one, maternity ward. At the latter, the proportion of non-Western women was 12%. The organization of targeted care did, therefore, not seem to be a solution at places with high immigrant density, but rather a practice where the density was lower than the national average.

The selection of immigrant women to the targeted ANC differed between the facilities: At three facilities, the targeted service was mainly for women who could not speak Danish, however other women with an immigrant background could also be seen here if capacity allowed. At two other facilities, the targeted service was for all with non-Danish appearing names based on the assessment of the referring midwife, or if the GP in the woman’s file had indicated that she was an ethnic minority woman. Finally, one facility provided targeted services for women born in Somalia only.

The midwives in charge of the targeted service emphasized that immigrant women constitute a very heterogeneous group. The organization of targeted service allowed more time per woman and consequently more attention to the individual needs of each woman. The midwives expressed that they had more flexibility in organizing their schedule than in the routine ANC schedule, for example, it was possible to book women more frequently or to book more time per consultation. Some facilities spent more than the double time per pregnant immigrant woman (a total of 4.5 h per woman) than was the routine ANC for non-immigrant women (2 h per pregnancy).

All the midwives at the targeted facilities expressed that they found the communication with ethnic minority women regarding normal, as well as acute, bodily changes during pregnancy and delivery difficult. The midwives explained this by referring to ethnic minority women having different health perceptions than the midwives. The midwives shared the perception that they needed to give more attention and time to explain the Danish ANC system: what to expect of the midwives, where to go with which symptoms, and how to establish contact with visiting nurses (home visiting nurses focusing on the health of newborns). At the facility targeting Somali women, special attention was given to female genital cutting, fear of cesarean section, and social vulnerability. Here they conducted group sessions bringing together pregnant women and new mothers.

The midwives expressed that care provision for women, whom both had a different cultural background and language barriers, often left them with uncertainty regarding how much of their inputs were actually understood. The midwives highlighted the need for good communication and interpreter services. For example, it was mentioned that it was a very challenging and time-consuming process to obtain informed consent for recruitment to research activities.

Lack of health education material in other languages than Danish was a general challenge. Some of the midwives mentioned that it was difficult to stay updated on new materials and expensive to have folders available in many different languages, so they requested for one national service that could keep track of this. Midwives at one hospital had developed a webpage with around 150 different short health education videos regarding pregnancy, birth, and the first three months after birth, which were synchronized into English, Turkish, Arabic, Urdu, and Greenlandic [25]. The majority of the midwives responsible for the immigrant-targeted facilities did not know about this webpage, indicating a gap in knowledge sharing.

The midwives providing the immigrant-targeted services were all women, who had volunteered to this assignment. None of them had had special training in cultural competencies, and they characterized their care provision to be based on a ‘learning by doing’-principle. They requested more attention to, and coordination in, this field and an organizational structure for knowledge sharing with other midwives. At one administrative unit, the immigrant-targeted care was placed at four different locations, and at this unit, the midwives had biannual meetings to share experiences and support each other, which they highly appreciated.

### 3.2. The Universal ANC Model

At the majority of maternity wards, no immigrant-targeted ANC was provided (18 of 24). At these wards, the proportion of non-Western immigrants ranged from 5% to 24%. Several midwives expressed that it was a deliberate decision not to single out immigrant women, as the barriers these women may face was considered to be a result of social aspects rather than aspects related to immigration, and therefore that all women should be treated according to their individual needs within universal care. At several facilities, immigrant-targeted ANC was abandoned by the end of the 1990s, as the midwives felt this was a stigmatizing practice.

### 3.3. Delivery and Post-Partum Care

Regarding the delivery and post-partum period at the maternity ward, few initiatives targeting immigrants were identified. At one site, a health education leaflet in Arabic was available for distribution to the new parents, and at another site, pictograms describing the normal procedures at the nursing ward (e.g., screening test of the newborn, breastfeeding initiation) were used.

### 3.4. Interpretation Services in Maternity Care

According to the national recommendation for ANC, a GP was obliged to assess an immigrant woman’s need for interpretation at first ANC visit. All managing midwives expressed that interpretation services could be arranged according to the hospital (regional) regulations. The professional interpretation service could be in person, by telephone, or using video, however, several issues related to the interpretation services were mentioned. Often the GP did not assess the language proficiency of the women, and the midwives would then have to identify such needs, resulting in a delay in communication. At times family members did the interpretation. At several facilities it was not possible to book extra time to interpreted consultations, making it difficult to cover all elements of care, as the communication takes at least double time.

## 4. Discussion

Six of 24 maternity wards were providing immigrant-targeted ANC, and thus the majority of facilities did not. It was not the facilities with a high proportion of births by non-Western immigrant women that had organized immigrant-targeted care. The targeted service implied longer and more consultations, as well as increased attention to the individual needs of the women. At these six facilities, navigation in the health care system, supporting communication of body symptoms, and use of interpreter services were mentioned as crucial issues. Arguments for not providing immigrant-targeted care were that immigrant women had nothing more in common than non-immigrant women and that targeted care, therefore, was stigmatizing. 

### 4.1. Weaknesses of Immigrant-Targeted ANC

The selection mechanism for immigrant-targeted care is controversial. In our study, the selection of women to immigrant-targeted care followed different criteria, using their name, language skills, or country of origin as indicators of being in special need. The process of designating vulnerable groups can have unintended effects, positive as well as negative, for both users and providers. The vulnerable group can feel that their needs are recognized and therefore creating a starting point for improved communication and patient safety. However, the categorization can also negatively bias the health professional’s perception of this group and introduce stigma [22]. Link and Phelan define stigma as follows: “Stigma exists when elements of labeling, stereotyping, separation, status loss, and discrimination occur together in a power situation that allows them” [26]. From this perspective, it can be argued that midwives with the power of the health care organizations can legitimize that specific groups of immigrant women are labeled and separated, potentially leading to the establishment of stereotypes of immigrant women as for example being weak/having lack of body awareness/being very sensitive to pain. This is a process potentially leading to stigma and discrimination. 

Considering a woman’s name to be related to her need for health care is a position where the diversity of immigrant women is neglected. Selection based on country of origin indicates a perception that these women belong to a distinct group. For the Somali group, this might relate to the attention this group has been given regarding, for example, female genital cutting [27], fear and refusal of intervention during delivery [7], and health care providers’ perceptions about negative Somali attitudes [28]. However, using such findings from research to directly label and select all women from specific groups is problematic as such cultural practices are contextual. The anticipation that all Somali women are a homogenous group can be seen as an example of a misunderstood and static definition of culture leading to a cultural archetype, as written by Kleinman and Benson [10]. They emphasize that culture is dynamic and changes from setting to setting and over time. Culture is something we all have and they state: “Culture is a process through which ordinary activities and conditions take on an emotional tone and a moral meaning for participants” [10]. Culture shapes our thinking and all humans tend to stereotype, so what is needed, and can be defined as cultural competence, is that health care providers reflect on how their thinking is embedded in culture and they need to have in mind how this affects their behaviors when meeting people with other cultures, dynamically defined [12]. This we can call dynamic cultural competence.

Grouping and selecting women to targeted care according to their language skills is less problematic as the selection follows an individual assessment of each woman’s ability to engage in health care interactions.

### 4.2. Strengths of Immigrant-Targeted ANC

Nevertheless, targeted care also has benefits. The midwives from the immigrant-targeted consultations found the targeted model highly useful to ensure good communication, and thus identify and accommodate the individual needs of immigrant women. Especially, the increased time and flexibility were mentioned as paramount to meet the very heterogeneous needs of these women.

Dubbin et al. argued that cultural and social distance between health care providers and users will cause disparities in care provision if these disparities are not well addressed. Using the Bourdieu-inspired concept of cultural health capital, they analyzed how patient-centered health care is more easily obtained by patients who share socioeconomic position and cultural understanding with the health care providers [29]. If such inequalities in care provision are to be overcome, health care providers need to understand and reflect on their own preconceptions when interacting with patients, in order to facilitate good communication and interaction. One may argue that targeted ANC creates increased sensitivity of the midwives, so that the cultural health capital and needs of the immigrant women are better understood, valued, and included in the care provision and that this will lead to increased communication, trust, concordance, and hence better quality of care for immigrant women.

From this argument, it follows that in existing universally organized ANC, midwives, due to organizational barriers (lack of time, flexibility, and interpreter services), fail to see and understand the needs of the ethnic minority women. In Denmark, two specialized immigrant medical clinics have been established to provide case management and coordination for complex immigrant patients where the universal health system has given up [30]. According to the clinicians here, the competencies to handle the complexity of some immigrant patients are not available in the general, universal, health care system [31]. Therefore, we find it likely that universal ANC provided without specific attention to the barriers immigrant women may face, continued training of midwives in dynamic cultural competence, and sufficient time and flexibility during care provision will continue to result in suboptimal care for ethnic minority women.

### 4.3. Lack of Policy Implementation

The midwives highlighted a need for approved and updated health education materials in different languages, an issue also highlighted among European immigrant health researchers [32]. The midwives at targeted care facilities had volunteered for this assignment but had not received any specific training in cultural competence and increased professionalization with pre- or postgraduate training of midwives seems relevant [33,34]. Without these elements, the recommendation from the national ANC policy of a culturally sensitive approach is difficult to accomplish.

We found that interpretation in maternal health care was not always conducted with the use of professional interpreters or supplemented with extra time, which is in correspondence with the general picture of interpreter services within the Danish health care system [35]. However, this practice is against the national ANC policy [20], and inadequate interpretation has been shown to compromise communication and quality of care [36,37]. In 2018, new legislation on interpretation services was introduced. Immigrants now have to pay for interpreters after three years of residence in Denmark [38]. As a consequence, the most vulnerable patient groups among immigrants limit seeking health care or bring family members as interpreters. This has been heavily debated as clinicians argue that this directly affects the quality of care and ethical principles of, for example, informed consent and not using children as interpreters. The interpretation law clearly compromises the national ANC policy, and how midwives are to navigate these opposing messages is not clear. For pregnant women, the fee for interpretation during an ANC visit is 334 Danish Kroner and during delivery 1675 Danish Kroner. This will further limit the possibilities for midwives to meet the individual needs of the immigrant women.

### 4.4. Immigrant Density

Finally, it is interesting to consider why the facilities providing targeted care tended to have the lowest immigrant density. One explanation is that when meeting an immigrant woman is a rare event, procedures to deal with this event are then developed. Another explanation may be that the units with many immigrants find it too resource demanding to provide more extensive health care for up to 1:5 of their clients.

### 4.5. Study Strengths and Limitations

This study depended on the personal answers of the administrative midwives. Some midwives gave detailed answers while others were concise. This study mapped the organizational practice and analyzed perceptions of midwives, and it could be very interesting to further study how immigrant women themselves see the balance between the recognizing and acknowledging elements of targeted care and the stigmatization issues of the selection to targeted care.

Our definition of immigrant-targeted and universal ANC is novel and was chosen as it identified situations where women were actively selected to immigrant-targeted care, and therefore allowed analysis of the labeling and selection process as well as the midwives’ perceptions of the needs of immigrant women and the shortcomings of the universal ANC system. The definition could be problematic, as women should receive needs-based care within the universal Danish ANC system (differentiation of care). Some immigrant women will, for sure, receive care according to their needs within the universal system. However, if the communication barriers (including language skills) are not addressed, the differentiation of care in the universal model will not fully capture the vulnerability of some ethnic minority women, leaving their needs unaddressed. The midwives expressed that there was no monitoring of potential ethnic differences in the enrolment into differentiated care. This is problematic, as it is likely that the assessment of some immigrant women’s social and mental health needs are overlooked, as it has been suggested for refugee children in Denmark [39]. We want to encourage clinicians and leaders of health care organizations to engage in a discussion of how to improve the precision of screening tools of targeted or differentiated care and call for an approach where issues of ethnicity, socioeconomic position, gender, mental health, and language skills are jointly assessed.

## 5. Conclusions

The majority of ANC facilities in Denmark did not enroll immigrant women in targeted ANC. In the targeted models, midwives had better conditions for providing culturally competent care due to more time, increased flexibility, and more consistent interpreter services. The care provision was characterized by increased attention to individual needs, however, cross-cutting issues were: interpretation, interpersonal interaction, and communication regarding body awareness and how to navigate in the health care system. The selection of immigrant women to targeted care was based on problematic criteria (incl. names) and should be carefully considered to avoid stigma. It seems likely that the universal models of ANC overlooked the needs of vulnerable immigrant women. To reduce ethnic disparity in maternal and child health, there is a need to improve the screening tools for targeted and differentiated care and to improve the universal ANC in terms of the dynamic cultural competencies of midwives, interpreter services, and flexibility in care provision.

## Figures and Tables

**Table 1:** The national recommendation for timing of antenatal care contacts for women enrolled in standard antenatal care *.

Gestational Week	General Practitioner	Midwife	Screening
6–10	X		
8 + 0 to 13 + 6			Double test
11 + 0 to 13 + 6			Nuchal translucency ultrasound
13–15		X	
18			Malformation ultrasound
21		X	
25	X		
29		X	
32	X		
35		X	
37		X	
39		X	
(41)		X	

* Anbefalinger for Svangreomsorgen (Recommendations for the antenatal care). Danish National Health and Medicines Authority. Copenhagen 2013; X marks a contact between the individual woman and the health care providers.

**Table 2:** Maternity wards in Denmark 2012: number of births by maternal origin in 2010 and immigrant-targeted antenatal care (ANC).

	Danish origin	Western Immigrant	Non-Western Immigrant	Total Births above or below 3000	Immigrant-Targeted ANC
Place of birth	%	%	%		
1	81.4	5.6	13.0	Above	No
2	80.7	6.9	12.4	Above	Yes
3	83.8	3.9	12.3	Above	No
4	71.5	4.2	24.3	Above	No
5	85.2	3.7	11.1	Above	No
6	89.6	3.2	7.2	Above	Yes
7	87.0	4.0	9.1	Above	No
8	85.3	4.3	10.4	Above	No
9	88.7	3.0	8.3	Below	No
10	91.2	2.3	6.6	Below	No
11	89.1	3.8	7.0	Below	No
12	87.9	4.0	8.1	Below	No
13	91.9	3.0	5.1	Below	No
14	89.9	3.7	6.4	Below	No
15	89.6	2.6	7.8	Below	No
16	90.4	2.1	7.5	Below	No
17	85.9	6.9	7.2	Below	Yes
18	92.1	1.7	6.1	Below	Yes
19	91.7	2.2	6.1	Below	No
20	89.4	3.8	6.8	Below	Yes
21	90.7	1.8	7.5	Below	No
Homebirth	94.4	3.1	2.4	Below	
22	88.6	4.5	6.9	Below	No
23	88.2	5.0	6.8	Below	Yes
24	91.6	2.3	6.0	Below	No
Total	85.7	4.1	10.3	Below	

The total number of births in this table is 58,263. The total number of births in 2010 was 63,515, but births to descendants of immigrants, birthplaces not operating in 2012, and 87 deliveries with missing information on the place of delivery are not presented in the table.
